# Efficacy And Safety of Acupoint Catgut Embedding for Perennial Allergic Rhinitis: Protocol for a Randomized Clinical Trial

**DOI:** 10.2196/63933

**Published:** 2025-04-21

**Authors:** Zijie Cai, ChunXue Meng, Fei Wang, ChunZhi Tang, Jing Zhang, Qian Zhang, Bin Guo

**Affiliations:** 1 Guangdong International Clinical Research Center of Chinese Medicine Guangzhou University of Chinese Medicine Guangzhou China; 2 Ningxia Medical University Yinchuan City, Ningxia Hui Autonomous Region China

**Keywords:** acupoint thread embedding, catgut embedding, allergic rhinitis, loratadine

## Abstract

**Background:**

Allergic rhinitis (AR) is a noninfectious chronic inflammatory disease of the nasal mucosa characterized mainly by itching, sneezing, nasal congestion, and rhinorrhea. It is mediated by immunoglobulin E (IgE). AR is one of the most common allergic diseases globally, affecting 10% to 20% of the population worldwide, with some regions even reaching rates as high as 50%, posing a global health issue. The prevalence of AR has been increasing since the 1960s, with a significant increase in recent years. At present, modern medicine—including desensitization therapy, the use of antiallergic drugs, antihistamines, hormones, and other treatments—can improve symptoms or regulate the immune system. However, both short- and long-term efficacy remain limited, as symptoms often recur after treatment cessation, and long-term drug use is associated with toxicity and side effects.

**Objective:**

Acupoint catgut embedding (ACE) therapy is widely used to treat AR in China. ACE therapy has been reported to be effective in managing the symptoms of AR, but the evidence faces methodological limitations. Therefore, we designed a parallel-arm, randomized controlled, multicentered, placebo-controlled, and single-blinded trial to evaluate the efficacy and safety of ACE therapy for AR.

**Methods:**

This study will be a parallel-group, patient-blind, placebo-controlled randomized controlled trial conducted in the Third Affiliated Hospital of Sun Yat-sen University, Ningxia Chinese Medicine Research Center, and the Affiliated Hospital of Shanxi University of Traditional Chinese Medicine. The trial consists of a 4-week treatment period, along with a 3-month follow-up. After providing written informed consent, eligible participants will be randomized at a ratio of 1: 1 into one of 2 groups: the ACE group receiving treatment and the sham ACE group. Both groups will receive conventional loratadine treatment.

**Results:**

The funding began in January 2022. The study was initiated on February 1, 2025, and will end in February 2026. Patient recruitment has already started, and the study results will be released in March 2026.

**Conclusions:**

We expect that this research will provide important insights into the efficacy of ACE treatment for AR and generate robust data for the foundation of future research in this field.

**Trial Registration:**

Chinese Clinical Trial Registry ChiCTR2500095634; https://www.chictr.org.cn/historyversionpubEN.html?regno=ChiCTR2500095634

**International Registered Report Identifier (IRRID):**

PRR1-10.2196/63933

## Introduction

Allergic rhinitis (AR) is a noninfectious chronic inflammatory disease of the nasal mucosa characterized mainly by itching, sneezing, nasal congestion, and rhinorrhea. AR is mediated by immunoglobulin E (IgE). It is one of the most common allergic diseases, affecting between 10% and 20% of the population worldwide [1], with some regions even reaching rates as high as 50%, posing a global health issue. The prevalence of AR has been increasing since the 1960s [2], with a significant increase in recent years [3]. At present, modern medicine—including desensitization therapy, antiallergic drugs, antihistamines, and hormones—helps improve symptoms and regulate the immune system. However, the short- and long-term efficacy is limited, as symptoms often recur after treatment cessation, and long-term drug use is associated with toxicity and side effects [4]. Allergen-specific immunotherapy (AIT) is the only means of altering the natural immunological course of allergic diseases and achieving long-term remission. Pharmacological measures can suppress the immune response and ameliorate symptoms, but there is a risk of relapse soon after stopping these measures [5]. Currently, the main drugs for treating AR are H1 antihistamines, nasal steroid hormones, and leukotriene receptor antagonists. The control and safety of nasal symptoms have been verified; however, limitations remain in preventing the recurrence of AR [6]. Catgut implantation at the acupoints is a subtype of acupuncture, in which a catgut is embedded in the acupoint using a special needle. The catgut is absorbed by the tissue over a period of time. Therapeutic effects can be achieved by continuing stimulation caused by the catgut at the acupoint. Therefore, catgut implantation at the acupoint may be effective in treating some chronic diseases such as AR. Although acupoint catgut embedding (ACE) therapy has been used to treat diseases for thousands of years in China, there have been very few clinical trials strictly designed to verify the efficacy and safety of this treatment for AR [7]. It is necessary to obtain stronger evidence for catgut implantation at the acupoints for treating AR. Research on ACE therapy for AR primarily focuses on regulating cytokines, influencing neurotransmitters, and inhibiting immune molecules such as IgE. At the cytokine regulation level, acupoint embedding research concentrates on interventions targeting inflammatory factors and transforming factors. Inflammatory factors include interleukin-17(IL-17), interleukin-4 (IL-4), and interferon-gamma (IFN-γ), and transforming growth factors include transforming growth factor β1, among others [8-12]. ACE therapy intervenes in neurotransmitters mainly by participating in the release of various immunologically active substances, such as substance P. Research has found that embedding therapy can alleviate rhinitis-related symptoms by regulating substance P and nitric oxide levels [13-15]. The process begins with mechanical stimulation, followed by biological and chemical stimulation. The gradual softening, decomposition, liquefaction, and absorption of the catgut thread within the acupoint provides prolonged stimulation, reducing the likelihood of recurrence. Therefore, the treatment interval can be extended to once every 10 days, greatly improving patient compliance. Moreover, catgut thread, as an allogeneic protein stimulus, can better regulate the relative balance of the body's internal environment, enhance immune function and stress resistance, and reduce allergic reactions. Therefore, it is considered an excellent method for treating AR [16].

A recently published systematic review confirmed the effect of ACE therapy. However, it also pointed out that previous randomized controlled trials (RCTs) faced a variety of methodological limitations, including the absence of sample size calculation, inappropriate control groups, and multicenter RCTs and blind designs [17]. Therefore, it is necessary to conduct large-scale and rigorously designed RCTs to overcome the identified methodological issues. The primary objective of this study is to evaluate the efficacy of ACE therapy for AR, with a focus on reducing recurrence. Secondary objectives include assessing whether ACE therapy can (1) improve patients' quality of life and (2) change the dosage of the relief medication (RM).

## Methods

### Study Design

**Figure 1:**
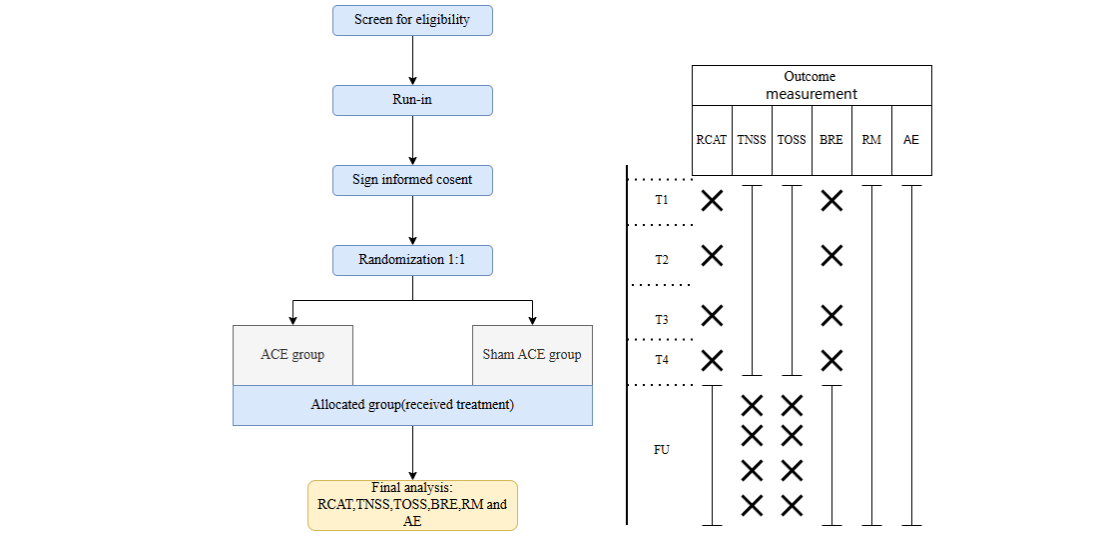
Trial flow diagram. ACE: acupoint catgut embedding; AE: adverse event; BRE: blood routine examination; FU: follow-up; RCAT: Rhinitis Control Assessment Test; RM: relief medication; TNSS: Total Nasal Symptom Score; TOSS: Total Ocular Symptom Score.

This study will be a parallel-group, patient-blind, placebo-controlled RCT conducted in the Third Affiliated Hospital of Sun Yat-sen University, Ningxia Chinese Medicine Research Center, and the future Affiliated Hospital of Shanxi University of Traditional Chinese Medicine. The flowchart of this trial is shown in Figure 1. The trial consists of a 4-week treatment conducted along with a 3-month follow-up. After providing written informed consent, eligible participants will be randomized at a ratio of 1: 1 into one of 2 groups: an ACE group receiving ACE treatment or a sham ACE group receiving sham ACE treatments. Both groups will receive conventional loratadine treatment.

### Patient Information

Participants will be recruited via advertisements, posters, leaflets about the trial, and doctor referrals from otorhinolaryngology clinics in the 3 study hospitals. Interested individuals will need to contact research assistants by phone or email. Trial information and consent forms will be sent to them to read before scheduling their first visit. In the first visit, screening evaluations will be conducted and recorded to ensure eligibility. For each participant who is eligible and willing to participate in the trial, research assistants will provide a signed consent form. The inclusion criteria for this trial are: (1) age between 18 and 65 years old; (2) meeting the diagnostic criteria for perennial AR; and (3) currently experiencing symptomatic attacks, able to accurately describe their condition, and willing to provide informed consent and participate in the clinical study.

Patients with AR were diagnosed based on the following criteria: (1) the presence of 2 or more symptoms including sneezing, clear watery nasal discharge, itchy nose, or nasal congestion that persists or occurs for more than 1 hour per day; (2) ocular symptoms such as itchy, watery, or red eyes; (3) nasal endoscopy showing pallor and edema of the nasal mucosa; and (4) a positive seasonal allergen test result for serum-specific IgE [18]. The exclusion criteria included: (1) recent respiratory infection or acute paranasal sinusitis within the past 14 days; (2) evidence of inflammation on chest X-ray; (3) history of chronic paranasal sinusitis or current diagnosis via X-ray examination; (4) the presence of organic lesions in the nasal cavity or recent nasal surgery; (5) diagnosis of paroxysmal respiratory diseases such as asthma; (6) recent use of H1-antihistamines, steroids, decongestants, or other medications affecting the respiratory system within the past 14 days; (7) specific immunotherapy or systemic hormone therapy within the last year; (8) recent use of acupuncture, moxibustion, cupping, nasal inhalation of traditional Chinese medicine, or other traditional therapies within the past 14 days; and (9) determination by clinical investigators that the patient is unable to adhere to the treatment regimen.

### Randomization and Allocation Concealment

A block randomization sequence was generated by SAS software (version 9.2; SAS Institute Inc), which was performed by Guangdong Provincial Hospital of Chinese Medicine’s Key Unit of Methodology in Clinical Research. Eligible participants will be randomly assigned to either the ACE group or the sham ACE group at a ratio of 1:1. An independent researcher will prepare treatment cards, on which a serial number and one of 2 names are printed, each representing one of the 2 groups. This independent researcher will be responsible for selecting and adding intestinal thread to the injection needles according to the respective groups. Treatment allocations will be stored in password-protected files and held independently by staff of the Key Unit of Methodology in Clinical Research. While receiving the first treatment, the participants will be given sequential treatment cards from independent researchers to ensure adequate concealment. The participants will be allocated into one of 2 groups according to the name printed on their treatment card.

### Blinding

The researchers will assign intervention measures to the participants according to their allocated groups. The participants will be blinded to their group allocations in this single-blind study, whereas the researchers and acupuncturists will be aware of the group allocations. To minimize bias, outcome assessors will be blinded throughout the study.

A first-level blind base will be established for data statistics, ensuring the statistician is unaware of the group assignments. All operations will be performed in accordance with established standard operation procedures. After each treatment session, the participants in the ACE and sham ACE groups will complete a questionnaire on whether they believe they received ACE treatment and how certain they are that active treatment was received on a 0-to-10 numeric rating scale, where 10 represents absolute certainty [18]. Both the block randomization and the blinding questionnaire will be exclusively administered by a single external party.

### Intervention

**Figure 2:**
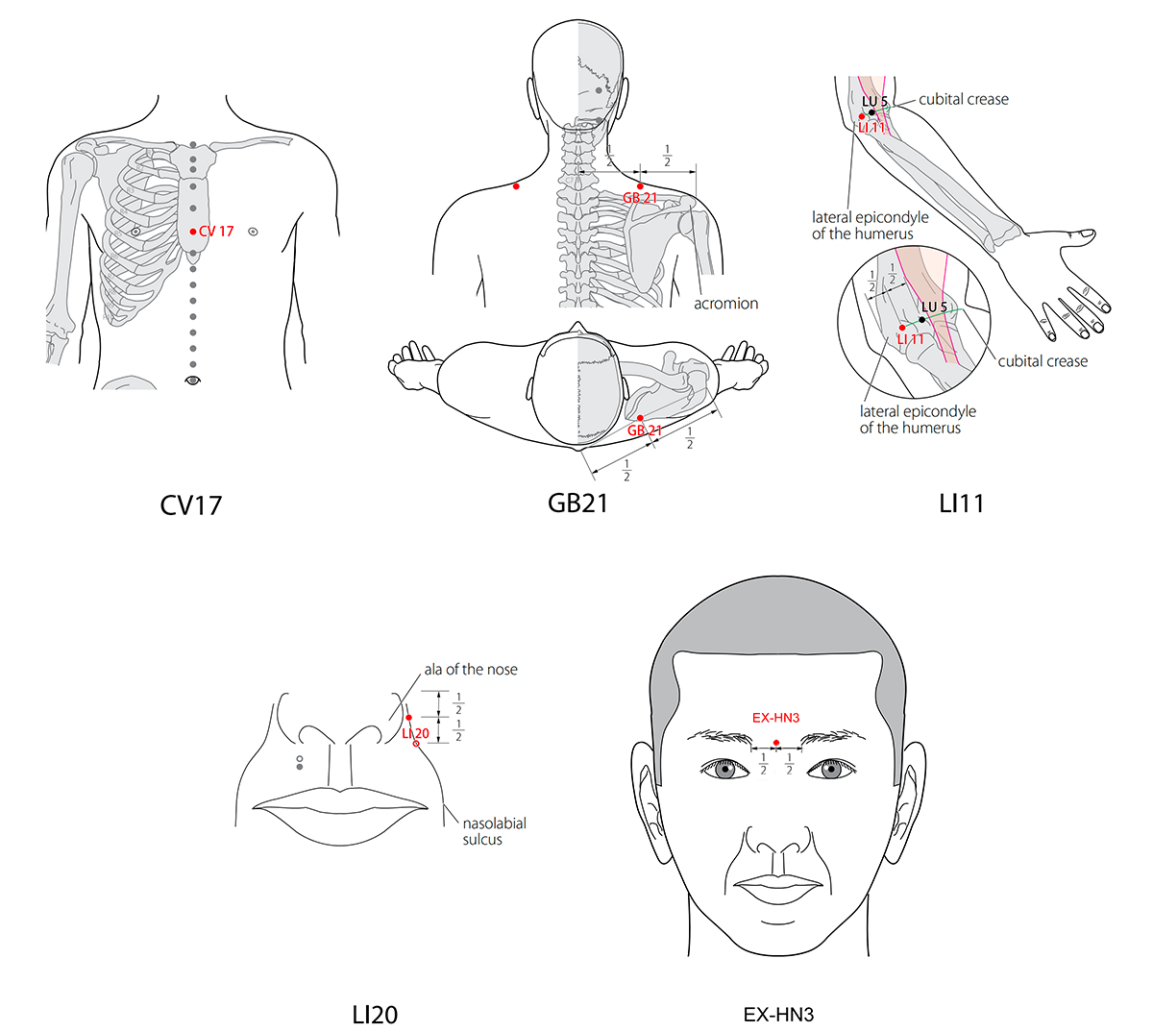
Diagram of the prescribed acupoints from the World Health Organization (WHO) 2014 Standard Acupuncture Point Locations in the Western Pacific Region (SPAR) study.

To ensure operational consistency, all patients will receive unified treatment set by the same doctor. The research assistant will add intestinal threads to the injection needles according to the respective groups. The ACE group will undergo ACE. The bilateral YingXiang (LI20), QuChi (LI11), JianJing(GB21), YinTang(GV24+), and DanZhong (CV17) points will be disinfected. Detailed positioning data are shown in Table 1 and Figure 2. The patient will assume a supine position, and acupoints will be located, marked with a marker, and disinfected with iodophor cotton swabs.

**Table 1:** Location of acupoints from the World Health Organization (WHO) 2014 Standard Acupuncture Point Locations in the Western Pacific Region (SPAR) study.

Acupoint	Location
YingXiang (LI20)	On the face, next to the midpoint of the outer edge of the alar, in the nasolabial groove
QuChi (LI11)	On the lateral aspect of the elbow, at the midpoint of the line connecting LU5 with the lateral epicondyle of the humerus
DanZhon (CV17)	In the anterior thoracic region, at the same level as the fourth intercostal space, on the anterior median line
JianJing (GB21)	In the posterior region of the neck, at the midpoint of the line connecting the spinous process of the seventh cervical vertebra (C7) with the lateral end of the acromion
YinTang (EX-HN3)	At the midpoint of the line connecting the 2 medial ends of the eyebrows

**Figure 3:**
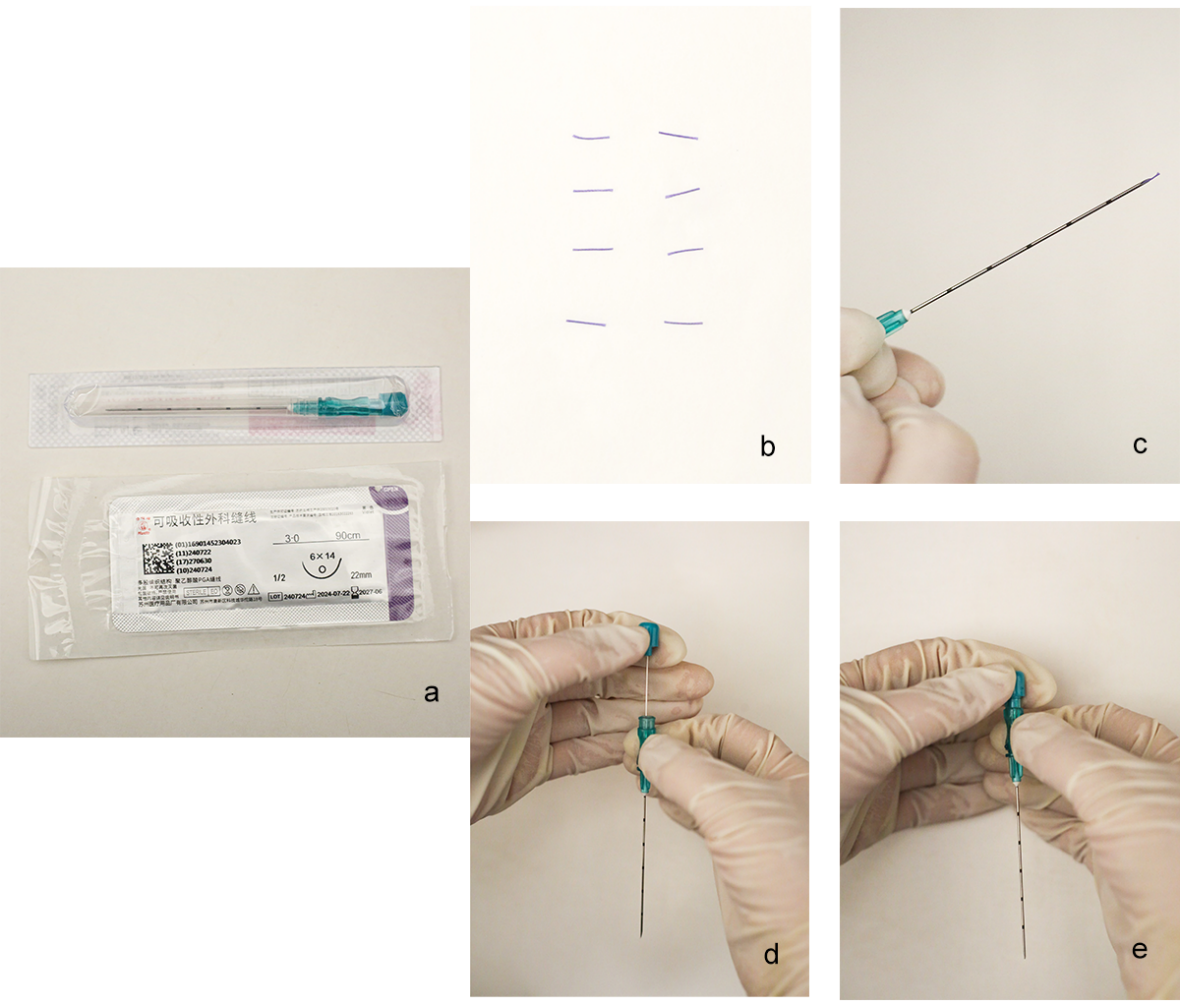
The process of acupoint catgut embedding (ACE) and the structure of the needle. a: Needles and absorbable surgical sutures. b: Specific cut-to-length. c: Insert the thread end into the needle eye. d: Insert the stylet into the needle. e: Insert the suture end into the acupoint.

The practitioner will wear aseptic gloves, cut the absorbable surgical suture around 5 mm, and place it into the front end of the needle tube. The structure of the acupuncture needle is illustrated in Figure 3. The doctor’s left thumb and index finger will tighten the skin around the operation site, and the needle will be inserted vertically with a catgut-burying needle in their right hand at a depth of 0.5 cm. The needle will be slightly lifted to stimulate the acupoints, and after getting qi, the left hand will push the needle core inward while the right hand withdraws the needle tube outward, embedding the surgical suture in the acupoint. After the stitches are placed, the pinhole will be pressed with aseptic gauze for a while and covered with a bandage or infusion paste.

Catguts will be implanted once every 7 days for a total of 28 days, and each participant will take a prescribed dose of loratadine orally every day. In the sham ACE group, participants will be placed in a supine position, and the acupoints will be located, marked with a marker, and disinfected with an iodophor cotton swab. The practitioner will wear sterile gloves and insert the needle into the skin. The doctor’s left thumb and index finger will tighten the skin around the operation site, and the needle will be inserted vertically with a catgut-burying needle in their right hand, at a depth of 0.5 cm. The needle will be slightly lifted to stimulate the acupoints, and after getting qi, the left hand will push the needle core inward. However, no absorbable surgical suture will be placed into the front end of the needle tube. After the procedure, the pinhole will be pressed with aseptic gauze for a while and covered with a bandage or infusion paste. This procedure will also be conducted once a week for 4 consecutive weeks. Each participant will take a prescribed dose of loratadine orally every day. Participants from both groups will be instructed to stop symptomatic RMs during the 1-week run-in and treatment periods. However, they will be allowed to take RMs if needed during the follow-up period. The use of RMs will need to be documented in the participants’ diaries.

### Outcome Measurement

For an evaluation of the primary and secondary outcome measures, the participants are required to complete 2 questionnaires, the Total Ocular Symptom Score (TOSS) and the Total Nasal Symptom Score (TNSS) [19]. These questionnaires will be completed at the beginning of each of the 4 treatment sessions (from week 2 to week 5) and in weeks 8, 12, and 16 during each follow-up session. During the follow-up process, the participants need to independently record their Rhinitis Control Assessment Test (RCAT) scores weekly [20]. In addition, the participants will be asked to complete diaries throughout the trial. The primary outcome is the recurrence rate 3 months after discontinuation. Once the treatment-effective participants discontinue the medication (RCAT score less than 21 points), they will immediately enter the follow-up period. If a relapse occurs, the relapse time will be promptly recorded, and the follow-up will conclude. Otherwise, the participants will continue to be monitored until 3 months after discontinuation of medication. RCAT is one of the most commonly used rhinitis control scales, and its reliability, validity, and responsiveness have been widely verified [21,22]. A score of 21 corresponds to the validated RCAT score of 25, which determines the controlled state of rhinitis. When RACT ≤21, it indicates an uncontrolled state of AR and the starting point for treatment [23]. RCAT is a simple self-rating scale that allows patients to assess their AR control at home and helps assess the success of long-term repeated treatment interventions [20,24]. TNSS evaluates 4 nasal symptoms: nasal obstruction, sneezing, rhinorrhea, and nasal itch. The symptoms are self-assessed and recorded by participants using a 5-point scale (0=no symptoms; 1=mild symptoms; 2=moderate symptoms; 3=severe symptoms; 4=very severe symptoms) [23]. TNSS scores range from 0 to 16, with low scores indicating lighter nasal symptoms. TOSS assesses eye symptoms, including redness, itchiness, and tearing. Each symptom is assessed on a scale between 0 and 3 (absent to most severe), with a total score from 0 to 9 [19,25]. The score represents an average for both eyes. The secondary outcomes include (1) changes in TNSS and TOSS score from baseline to weeks 8, 12, and 16; (2) response to interventions, defined as participants with a change in TNSS score of ≥0.5 from baseline; (3) and serum levels of tumor necrosis factor-alpha (TNF-α), IFN-γ, IL-4, IL-17, and IgE, measured by enzyme-linked immunosorbent assay according to the manufacturer's protocol (Bio-Techne).

### Serum Acquisition

The participants’ venous blood samples will be collected using blood collection tubes, symmetrically placed in the centrifuge sample bin, and covered. The rotation speed will be set to 4000 rpm, and the centrifuge time will be 10 minutes. The machine will be turned off after centrifugation, the sample bin opened, the sample carefully taken out, and the separation of the sample observed. If it is unsatisfactory, the sample will be centrifuged again. The serum will be carefully sucked from the upper layer with a 1-ml pipette, moved to an Eppendorf tube, and stored in a refrigerator at –80 °C degrees for inspection. Serum TNF-a, INF- γ, IL-17, IL-4, and IgE levels will be measured using enzyme-linked immunosorbent assay [26].

### Basic Characteristic Variables

Demographics such as age, sex, height, body mass, and BMI will be obtained. Vital signs including temperature, blood pressure, heart rate, and breathing will also be measured. Data on patient medical history, family history, risk factors, and dietary history will be collected.

### Statistical Analyses

Intentional therapeutic analysis will be adopted, with missing values estimated using the last observation carried forward method. Mean and SD (for continuous variables that obey normal distribution), median and quartile (for continuous variables that do not obey normal distribution), and frequency and percentage (for classified variables) will be used for the statistical description. According to the numerical characteristics of the variables, quantitative data such as age, height, and weight of the 2 groups will be compared using a *t* test or the Wilcoxon rank sum test. The chi-square test or Fisher exact probability method will be used to compare qualitative variables, such as sex and nationality between the 2 groups. Cox regression analysis will be carried out with recurrence outcome and time of first recurrence as dependent variables, and the differences in survival curves between both groups will be compared. SPSS software (version 23.0; IBM Corp) will be used for statistical analysis. A 2-sided test will be used for all statistical inferences, and *P*<.05 will be considered statistically significant.

### Safety Assessment

An independent data and safety monitoring committee evaluates the progress of the trial and assesses the safety data that may be requested during the trial. An adverse event (AE) is defined as any undesirable experience that participants undergo during the trial period, whether it is associated with the intervention or not. Participants will be instructed to report any AEs to the research team at any time. All details of AEs such as time of occurrence, severity, management, and causality to the intervention will be recorded on case report forms (CRFs). There are nine common AEs related to ACE: (1) lingering discomfort; (2) local hematoma or subcutaneous bleeding; (3) local swelling; (4) local induration; (5) severe pain; (6) thread extrusion; (7) local itching; (8) elevated body temperature after embedding the thread; and (9) local redness, swelling, heat, and pain after embedding the thread [27]. All AEs will be followed up from the date they are brought to the investigator’s attention until the AE is resolved. In the event of an emergency medical situation, the individual’s randomization code and group allocation will be identified via a standard operational procedure.

### Quality Control

Before recruitment, the entire research team including acupuncturists, operational assistants, and research nurses were required to attend a training workshop. This was done before the trial to ensure their strict adherence to the study protocol and familiarity with the trial administration process. They were also provided with a written protocol and standard operation procedure documents. The 2 acupuncturists who will administer the treatment each have acupuncture licenses from the Chinese Ministry of Health and have over 5 years of experience in ACE application. The data collected in this trial comprise information recorded in CRFs and information on the RCAT, TNSS, TOSS, and blood routine examinations. Data will be entered using the double-entry method. The data quality will be checked regularly by research assistants and overseen by monitors. Data monitoring will be conducted regularly following standard operation procedures set by the Guangdong International Clinical Research Center of Chinese Medicine (Guangzhou, China). Audits will be performed regularly by the Department of Science Research at the Guangzhou University of Chinese Medicine. All modifications will be marked on the CRFs. Data managers will then recheck the data before logging them and promptly notify the research team if any discrepancy is found. The database will be locked after all data have been cleaned. If participants withdraw from the trial either during the treatment period or the follow-up phase, they are not required to provide any reasons. The withdrawal rate will be analyzed via statistics.

### Sample Size Calculation

Our preliminary study [28] indicated that the 3-month recurrence rate of the ACE group should be at least 5% lower than that of the Sham ACE group to be clinically significant. Using α=0.025 (unilateral), β=0.2, △=5%, and K=1, the sample size was estimated based on the comparison of two sample rates in the optimization test. The sample size in each group was calculated to be 52. Since the effectiveness of any treatment was not 100%, patients with ineffective treatment in this study could not be included in the assessment of recurrence, so the loss of follow-up rate in this study was 20%, which amounted to 126 cases. The \following equation is used to calculate the sample size, which refers to the estimated number of patients to be enrolled in the study:



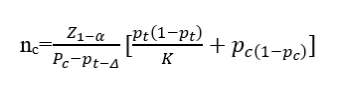



### Ethical Considerations

This study protocol has been approved by the Ethics Committee of the Guangzhou University of Chinese Medicine (B2014-014-01). It is explicitly outlined to all participants that the trial involves a waiting-list group and that informed consent to participate includes an agreement to undergo 3 months of clinical monitoring. All participants are provided with enough time to decide whether to sign the informed consent. This clinical study will adhere to the ethical principles outlined in the Declaration of Helsinki (2024) and relevant national regulations. Prior to study initiation, the trial protocol underwent rigorous review and approval by the Ethics Committee of Ningxia Medical University (2024-075). Written informed consent must be obtained from each participant before randomization.

## Results

The study funding began in January 2022. The study was initiated on February 1, 2025, and will end in February 2026. Participant recruitment is underway, and the results will be released in March 2026.

## Discussion

Presently, there are many limitations in the treatment of AR. The main drugs for treating AR are H1 antihistamines, nasal steroid hormones, and leukotriene receptor antagonists. The control and safety of nasal symptoms have been verified, but there are still deficiencies in controlling the recurrence of AR [6]. AIT is the only means of altering the natural immunological course of allergic diseases and achieving long-term remission. Pharmacological measures can suppress the immune response and/or alleviate the symptoms, but there is a risk of relapsing soon after these measures are withdrawn. The current AIT approaches depend on the administration of intact allergens, often comprising crude extracts of the allergen. In contrast, ACE has many advantages, involving mechanical stimulation and later biological and chemical stimulation [29]. The process of softening, decomposing, liquefying, and absorbing the catgut thread within the acupoint generates long-lasting stimulation, making it less prone to recurrence. Therefore, the treatment interval can be extended to once every 10 days, greatly improving patient compliance. Moreover, the catgut thread, as an allogeneic protein stimulus, can better regulate the relative balance of the body's internal environment, enhance immune function and stress resistance, and reduce allergic reactions [16]. Currently, the research on ACE therapy for AR primarily focuses on regulating cytokines, influencing neurotransmitters, and inhibiting immune molecules such as IgE. At the cytokine regulation level, acupoint embedding research concentrates on interventions targeting inflammatory factors and transforming factors. Inflammatory factors include IL-17, IL-4, and IFN-γ [30], and transforming growth factors include transforming growth factor β1, among others [8-12]. ACE therapy intervenes in neurotransmitters by participating in the release of various immunologically active substances, such as substance P. Research has found that embedding therapy can alleviate rhinitis-related symptoms by regulating substance P and nitric oxide levels [13-15]. Importantly, a study found that ACE therapy decreased the release of immunoglobulin G (IgG) in vivo in mice, which was accompanied by a decrease in IgG1, histamine, and interleukin. The symptoms of AR in mice were likewise alleviated during this process [25].

The purpose of this trial is to reduce the recurrence of rhinitis, relieve symptoms, and achieve clinical benefit by using ACE as a nondrug therapy. Previous articles about ACE for the treatment of AR lack placebo-controlled, high-quality research [26]. Therefore, it is necessary to provide high-quality research in this domain. We designed this RCT to examine whether ACE treatment decreases recurrence and improves symptoms such as sneezing, clear watery nasal discharge, itchy nose, and nasal congestion in patients with AR. The main limitation of this study is its single-blind design. Another potential limitation is the possibility of patient attrition due to the requirement of undergoing treatment over 4 weeks, totaling 4 times. Another limitation is our inability to design double-blind trials due to the material and design of the embedding needles. Moving forward, our aim is to develop improved embedding needles that align more closely with the requirements of double-blind testing, thereby enhancing the international promotion of this therapy. The strengths of this study include the use of a randomized controlled design, with all patients being followed up. Both subjective and objective data will be collected in this study to examine the effectiveness of ACE in treating AR. Another advantage is that it focuses on decreasing recurrence. The results of this study could improve the treatment outcomes of patients with AR. We posit that this research will provide important insights into the efficacy of ACE treatment for AR and generate robust data for the foundation of future research in this field.

## Abbreviations

ACE: acupoint catgut embedding
AE: adverse event
AIT: allergen-specific immunotherapy
AR: allergic rhinitis
CRF: case report form
IgE: immunoglobulin E
IgG: immunoglobulin G
INF-γ: interferon-gamma
IL-17: interleukin-17
IL-4: interleukin-4
RCAT: Rhinitis Control Assessment Test
RM: relief medication
TNF-α: tumor necrosis factor-alpha
TNSS: Total Nasal Symptom Score
TOSS: Total Ocular Symptom Score

## Data Availability

Data sharing is not applicable to this article as no data sets were generated or analyzed for this article. The data sets analyzed during this study will be available from the corresponding author upon reasonable request.

## References

[1] Brozek JL, Bousquet J, Baena-Cagnani CE, Bonini S, Canonica GW, Casale TB, van Wijk RG, Ohta K, Zuberbier T, Schünemann HJ, Global AllergyAsthma European Network, Grading of Recommendations Assessment‚ DevelopmentEvaluation Working Group (2010). Allergic Rhinitis and its Impact on Asthma (ARIA) guidelines: 2010 revision. J Allergy Clin Immunol.

[2] Latvala J, von Hertzen L, Lindholm H, Haahtela T (2005). Trends in prevalence of asthma and allergy in Finnish young men: nationwide study, 1966-2003. BMJ.

[3] Ahmadiafshar A, Maarefvand M, Taymourzade B, Mazloomzadeh S, Torabi Z (2012). Efficacy of sublingual swallow immunotherapy in children with rye grass pollen allergic rhinitis: a double-blind placebo-controlled study. Iran J Allergy Asthma Immunol.

[4] Deng G, Wan Q, Wang J, Ye W (2020). Efficacy and safety of Tian moxibustion in treating allergic rhinitis: A protocol for a systematic review and meta-analysis. Medicine (Baltimore).

[5] Wraith DC, Krishna MT (2021). Peptide allergen-specific immunotherapy for allergic airway diseases-State of the art. Clin Exp Allergy.

[6] Liao C, Liu T, Zeng Z, Wang D, Tang G, Wang H, Tian L (2020). Efficacy and safety of modified Yupingfeng formula in treating allergic rhinitis: A protocol for systematic review and meta analysis. Medicine (Baltimore).

[7] Li X, Zhang Q, Jiang L, Li T, Liu M, Liu H, Wang X, Zhang F (2013). Clinical effect of catgut implantation at acupoints for allergic rhinitis: study protocol for a randomized controlled trial. Trials.

[8] Ouyang Y, Miyata M, Hatsushika K, Ohnuma Y, Katoh R, Ogawa H, Okumura K, Masuyama K, Nakao A (2010). TGF-β signaling may play a role in the development of goblet cell hyperplasia in a mouse model of allergic rhinitis. Allergology International.

[9] Knipping S, Holzhausen HJ, Riederer A, Schrom T (2009). Allergic and idiopathic rhinitis: an ultrastructural study. Eur Arch Otorhinolaryngol.

[10] Maes T, Tournoy KG, Joos GF (2011). Gene therapy for allergic airway diseases. Curr Allergy Asthma Rep.

[11] Zhang H, Phan SH (1999). Inhibition of myofibroblast apoptosis by transforming growth factor beta(1). Am J Respir Cell Mol Biol.

[12] Li Y, Zhou F, Zhang H, Chen X, Yin M, Tang M, Zhang J, Lv Z, Yang H, Huang Y, Li X (2024). Acupoint catgut embedding therapy in traditional Chinese medicine for managing allergic rhinitis. J Vis Exp.

[13] Venge P (2003). Monitoring the allergic inflammation. Allergy.

[14] Heyin H, Yichen W, Qinxiu Z, Xiaojuan WU, Jiang W, Xiangyu L, Rui P, Li F, Liu C, Luo T (2023). Efficacy of catgut embedding in Baihui (GV20) and Feishu (BL13) and Pishu (BL20) on lung tissue, brain tissue and blood related indexes in rats with allergic rhinitis of lung deficiency type. J Tradit Chin Med.

[15] Tang M, Wang J, Zhang Q (2024). Clinical efficacy of acupoint catgut embedding in the treatment of allergic rhinitis: A systematic review and meta-analysis. Am J Otolaryngol.

[16] Xiang F, Zhang H, Jing R, Zheng J, Zhang J, Zhang Q, Li X (2024). Yingxiang acupoint embedding improves mucosal barrier function in rats with local allergic rhinitis. Int Arch Allergy Immunol.

[17] Zhang Y, Qi L, Wang R (2023). Meta-analysis: reducing the recurrence rate of allergic rhinitis through oral administration of traditional Chinese medicine. Eur Rev Med Pharmacol Sci.

[18] Leth-Møller KB, Skaaby T, Linneberg A (2020). Allergic rhinitis and allergic sensitisation are still increasing among Danish adults. Allergy.

[19] Choi SM, Park J, Li S, Jung H, Zi M, Kim T, Jung S, Kim A, Shin M, Sul J, Hong Z, Jiping Z, Lee S, Liyun H, Kang K, Baoyan L (2013). A multicenter, randomized, controlled trial testing the effects of acupuncture on allergic rhinitis. Allergy.

[20] Nathan RA, Dalal AA, Stanford RH, Meltzer EO, Schatz M, Derebery J, Mintz M, Thompson MA, Dibenedetti DB (2010). Qualitative development of the Rhinitis Control Assessment Test (RCAT), an instrument for evaluating rhinitis symptom control. Patient.

[21] Meltzer EO, Schatz M, Nathan R, Garris C, Stanford RH, Kosinski M (2013). Reliability, validity, and responsiveness of the Rhinitis Control Assessment Test in patients with rhinitis. J Allergy Clin Immunol.

[22] Liedtke J, Mandl A, Köther J, Chwieralski J, Shah-Hosseini K, Raskopf E, Pieper-Fürst U, Allekotte S, Mösges R (2018). RCAT reflects symptom control and quality of life in allergic rhinoconjunctivitis patients. Allergy.

[23] Wang Y, Chen H, Zhu R, Liu G, Huang N, Li W, Yang L, Zhang S, Qi S, Daurès J, Chiriac AM, Demoly P (2016). Allergic Rhinitis Control Test questionnaire-driven stepwise strategy to improve allergic rhinitis control: a prospective study. Allergy.

[24] Chaibi A, Šaltytė Benth J, Bjørn Russell M (2015). Validation of placebo in a manual therapy randomized controlled trial. Sci Rep.

[25] Gödicke V, Hundt F (2010). Registration trials for specific immunotherapy in Europe: advanced guidance from the new European Medical Agency guideline. Allergy.

[26] Hou Y, Wang D, Zhou S, Huo C, Chen H, Li F, Ding M, Li H, Zhao H, He J, Da H, Ma Y, Qiang Z, Chen X, Bai C, Cui J, Gao N, Liu Y (2024). Probiotics combined with prebiotics alleviated seasonal allergic rhinitis by altering the composition and metabolic function of intestinal microbiota: a prospective, randomized, double-blind, placebo-controlled clinical trial. Front Immunol.

[27] Wang X, Lin G (2020). Analysis of adverse reactions of ACE therapy. Chin Acupunct Moxibust.

[28] Yin Zihan, Geng Guoyan, Xu Guixing, Zhao Ling, Liang Fanrong (2020). Acupuncture methods for allergic rhinitis: a systematic review and bayesian meta-analysis of randomized controlled trials. Chin Med.

[29] Wang YF (2023). 31 cases of AR treated by acupuncture combined with ACE. Chin Acupunct Moxibust.

[30] Li X, Zhang Q, Liu M, Chen Q, Liu Y, Zhang F, Deng J, Zhong Z (2014). Catgut implantation at acupoints for allergic rhinitis: a systematic review. Chin J Integr Med.

